# Removal of the Alveolar Processes of Both Jaws

**Published:** 1826-07

**Authors:** 


15. Dr. Regnoli of Forli, relates a case, in which a fungoid affection of the maxillæ and gums was successfully treated by the removal of the alveolar processes of both jaws. The patient, a woman thirty-five years of age, had had carious teeth from her infancy, and was almost constantly tormented with severe tooth-ache. She was besides subject to frequent erysipelas of the head and neck.

Towards the close of 1824, she discovered a small tumour behind the last molar tooth of the lower jaw on the right side. It soon ulcerated, and rapidly spread to the gums and alveoli of both jaws. These parts were much swollen, and considerably contracted the cavity of the mouth. The fungoid excrescences poured out blood on the slightest touch, and continually produced a thin and fetid discharge. The deformity was considerable, and the voice was altered. The limits of the disease were well defined, and the lymphatic system did not appear to be affected, but the patient experienced much pain, her countenance was dull and cachectic, she lost flesh, and had febrile exacerbations in the evening. In this state of things, the patient was admitted into the hospital at Pesaro, where after having first performed the operation on the dead subject, Dr. Regnoli removed the teeth and alveolar processes of both jaws, with the exception of the last molar tooth on the left side of the lower jaw, the socket of which appeared to be sound. From the situation of the parts the saw could hardly be employed, hence it was merely used to form a shallow groove in the most prominent parts of the bone, the separation of which was effected by means of a chissel and mallet. Actual cautery was applied to the bleeding vessels, and to such suspicious parts as were not accessible to the knife. The lips of the external wound were brought together by three gold needles and the twisted suture.

The first day after the operation, the patient referred her pain to the throat, rather than to the parts which had been operated upon. She had severe headache, which was attributed in part to the shock given to the head by the strokes of the mallet, and to the division of the dental nerves. The needles were removed on the fifth day. On the fifteenth, seventeenth, and eighteenth, some portions of exfoliated bone were detached—on the nineteenth, the lips could be closed for the first time. By the twenty-third all tumefaction had subsided, the voice was improved, the catamenia, which had long been absent, had re-appeared, and the other functions were in a natural state. On the thirtieth the sole remaining tooth was removed, as it interfered with mastication. Five days later, she left the hospital in good health. The lips fell in a little, especially the lower, but the deformity was very slight. The voice, which had not quite recovered itself, was daily improving.

Dr. Regnoli concludes, that though the disease should return, the operation was still proper and necessary. Without it, he considers that death would have been inevitable, and he urges in its favour—that it incurred but little danger—that the practice of Dupuytren and Vacca support it—and that the disease does not always return.

